# Chromatographic analysis of the chemical composition and anticancer activities of *Curcuma longa* extract cultivated in Palestine

**DOI:** 10.1515/biol-2022-0767

**Published:** 2023-11-23

**Authors:** Murad Abualhasan, Nidal Jaradat, Mohammed Hawash, Naser Shraim, Mohammad Asaad, Ahmed Mousa, Zain Mousa, Reem Tobeh, Balsam Mlitat

**Affiliations:** Department of Pharmacy, Faculty of Medicine and Health Sciences, An-Najah National University, Nablus, 00970, Palestine; Department of Biomedical Sciences, Faculty of Medicine and Health Sciences, An-Najah National University, Nablus, 00970, Palestine

**Keywords:** *Curcuma longa*, curcumin, cytotoxicity, plant, chromatography, mass spectrometry

## Abstract

*Curcuma longa* (turmeric) is a plant that has been extensively utilized in traditional medicine for centuries. Turmeric has a long history of use in both food and traditional medicine for the treatment of ailments such as diarrhea, cancer, flatulence, and dyspepsia. In Palestine, this plant was cultivated for the first time. The objective of this study was to characterize the extract of *C. longa* and assess its antimutagenic activity against a variety of cancer cells. Gas chromatography–mass spectrometry (GC–MS) and high-performance liquid chromatography (HPLC) methods were employed to identify the constituents of turmeric. The cytotoxic effects of *C. longa* were evaluated on cancer and normal cell lines using the 3-(4,5-dimethylthiazol-2-yl)-5-(3-carboxymethoxyphenyl)-2-(4-sulfophenyl)-2*H*-tetrazolium assay. The results revealed the presence of 10 components in turmeric extract as identified by GC–MS. The major constituents comprising 78% of the total constituents were α-zingiberene (27.51%), tumeron (19.44%), β-sesquiphellandrene (19.40%), and aromatic-tumeron (11.63%). HPLC analysis successfully separated the main constituent, curcumin (1.78%), along with two other curcumin derivatives. The cytotoxicity results demonstrated potent anticancer activity of the *C. longa* extract against HeLa and LX2 cell lines, with IC_50_ values of 46.84 ± 2.12 and 29.77 ± 1 µg/mL, respectively. Furthermore, the plant extract at a concentration of 250 µg/mL exhibited over 95% inhibition against all tested cancer cell lines. These findings highlight the promising potential of turmeric as a natural source with powerful anticancer activities. Moreover, the extract may possess other biological activities such as antioxidant and antimicrobial properties, which could be explored in future studies.

## 1 Introduction

The use of medicinal plants in traditional medicine has been vital for the treatment of ailments since ancient times [1]. The induction of cytotoxicity is one of the most important signs of anticancer medicines. People with cancer regularly utilize herbal therapies or herbal supplements, according to several surveys [2]. Turmeric (*Curcuma longa* L.) is a perennial rhizomatous herbaceous plant in the Zingiberaceae family, native to South Asia [3]. It is commonly cultivated as a spice and medicinal herb in tropical and subtropical regions around the world [4]. While turmeric is mostly grown as a commercial crop, it can also grow wildly in certain regions [5].

*C. longa* is a tropical herb and requires a warm and humid climate to grow. It thrives in well-draining soil and requires a lot of rainfall or irrigation to grow successfully [6].

Phytochemical components of *C. longa* in various tissues have been investigated extensively. Since its discovery, at least 235 phytochemicals, mostly terpenoids and phenolic molecules, have been identified from this herb, e.g., 22 diarylheptanoids and diarylheptanoids, 2 alkaloids, 4 sterols, 3 triterpenoids, more than 100 sesquiterpenes, 5 diterpenes, 68 monoterpenes, 8 phenylpropene and other phenolic molecules, and 14 other compounds [7].

Diarylheptanoids (curcuminoids) and essential oils (EOs) are the two major classes of bioactive constituents in C*. longa* rhizomes, with a wide range of *in vivo* and *in vitro* assay bioactivities [8]. Curcuminoids are predominantly found in *C. longa* rhizomes [9]. Monoterpenes predominate in EOs extracted from flowers and leaves, whereas sesquiterpenes predominate in EOs extracted from rhizomes and roots [10,11]. Although there are considerable differences in the composition of the EOs of *C. longa* rhizomes with varieties and geographic regions, the concentrations of curcuminoids in *C. longa* rhizomes fluctuate often with cultivation conditions, sources, locations, and varieties [12,13].

Moreover, the quantities of curcuminoids and EOs vary depending on the extraction technique used, and both are unstable throughout the extraction and storage phases. As a consequence, there is a wide range in quality among commercialized *C. longa* product offerings [14].

While curcumin, demethoxycurcumin, and bisdemethoxycurcumin have been utilized as biomarkers for the quality control of extracts, powders, and *C. longa* rhizomes products, Ar-turmerone, α-turmerone, and β-turmerone are utilized for the quality control of *C. longa* oleoresin and EO products [7].

However, there are a number of modifiable risk factors, such as smoking and drinking alcohol intoxicatingly [15].

Therefore, the current investigation aims to identify the chemical components of *C. longa* rhizome extract cultivated in Palestine for the first time using gas chromatography–mass spectrometry (GC–MS) and high-performance liquid chromatography (HPLC) techniques and estimate their cytotoxic effect using an 3-(4,5-dimethylthiazol-2-yl)-5-(3-carboxymethoxyphenyl)-2-(4-sulfophenyl)-2*H*-tetrazolium (MTS) assay.

## 2 Material and methods

### 2.1 Plant material

The *C. longa* plant rhizomes were harvested from Sarra Turmeric farm located in Nablus city, Palestine in November 2022. The characterization of the plant species was conducted in the Pharmacognosy Laboratory at An-Najah National University, and the plant sample was deposited in the same laboratory with a voucher specimen code of Pharm-PCT-2709.

The fresh rhizomes were cleaned well with running tap water for 30 min and rinsed with distilled water several times until they were finally cleaned well. The clean rhizomes were chopped into small pieces, and 235.34 g of the plant material was placed in a 1 L glass bottle and macerated for 5 days with 500 mL dichloromethane (DCM) solvent. Then, the mixture was filtered. The filtrate was left in the fume hood for the evaporation process of DCM for 1 week. After that, the extract resulting from evaporation was transferred into a container by dissolving the extract with a small amount of hexane for ease of transportation and then left without a cover to evaporate for 2 days [16]. The *C. longa* plant rhizomes DCM extract yield was 4.46%.

### 2.2 GC–MS assessment

The GC–MS techniques were used to identify *C. longa* plant rhizomes DCM extract, which was examined by a Perkin Elmer Clarus 500 gas chromatograph with a Perkin Elmer Clarus 560 mass spectrometer. SLB^TM^-5ms fused-silica capillary column (30 m × 0.25 mm, film thickness 0.25 µm) was utilized to perform the separation. The temperature of the oven, including the column, was set to rise by 4°C every minute, beginning at 50°C and ending at 280°C. Helium was used as a carrier gas at a constant flow rate of 1 mL/min during the whole chromatographic run. At a temperature of 250°C, 1 µL of the tested extract was dissolved in methanol and then injected in split mode with a split ratio of 1:50. The National Institute of Standards and Technology’s MS Data Center reference spectra were compared to the mass spectra of the chemical components, and their Kovats retention indices were compared to values given in the literature. The Kovats Retention Index for each compound was calculated using the retention time value from the Hydrocarbon Alkane Standard [17].

### 2.3 HPLC analysis

#### 2.3.1 HPLC conditions

The HPLC technique was used to analyze the curcumin and curcuminoids in *C. longa* plant rhizomes DCM extract. The reverse phase chromatography analytical method was performed using Binary HPLC Pump Waters 1525 with a six-port manual injector and a Waters 2998 diode array detector by 425 nm (for curcuminoids) for chromatograms, and Breeze 2 software was utilized for instrument control, data collection, and data processing. The mobile phase was an isocratic combination of acetonitrile 50% and water 50% with a flow rate of 1 mL/min. The injection volume for all samples and standard solutions was 20 µL. The column Symmetry Shield™ RP-18 5 µm 4.6 × 250 mm was used.

#### 2.3.2 HPLC procedure

##### 2.3.2.1 Sample preparation

About 87 mg of *C. longa* plant rhizome extract was transferred into a 10 mL volumetric flask. Then, add 6 mL of methanol and sonicate while stirring to dissolve the mixture, then complete the volume with methanol. The small portion was filtered through a 0.45 µm syringe filter and injected into the HPLC system.

##### 2.3.2.2 Standard preparation

A stock solution (1000 mg/mL) was prepared by weighing an equivalent quantity of standard curcumin. The weighed substance was then placed into a 100 mL volumetric flask, followed by the addition of 60 mL of methanol. After sonication and thorough mixing, the solution was finally brought up to a total volume of 100 mL using methanol. Filter the portion through a 0.45 µm membrane. To prepare the standard (0, 50, 150, 200, 250, 300 µg/mL), add 0.5, 1, 1.5, 2, 2.5, and 3 mL of stock standard to six volumetric flasks (100 mL). Add 60 mL methanol and sonicate and mix to dissolve the mixture, complete the volume, and then inject the standards.

### 2.4 Cytotoxicity method

RPMI 1640 medium was used as a culture medium to grow skin tumors (B16-F1), colorectal adenocarcinoma (COLO 205, Caco-2), cervical adenocarcinoma (HeLa), and human hepatic stellate (LX-2) with 1% l-glutamine, 1% penicillin/streptomycin, and 10% fetal bovine serum added. The cells were cultured in a humidified environment at 37°C with a 5% CO_2_ atmosphere, and a 96-well plate was then used to seed the cells at 5 × 10^3^ cells/well. After 48 h cells were incubated with various concentrations (10, 50, 100, 500, and 700 µg/mL) of the tested aromatic oil and Doxorubicin for 24 h. According to the package recommendations, the Cell-Tilter 96® Aqueous One Solution Cell Proliferation (MTS) bioassay (Promega Corporation, Madison, WI) was used to measure the cell viability. After the treatment, 20 μL of MTS solution per 100 μL of media was added to each well, and the plates were incubated for 2 h at 37°C. The absorbance was measured using a ultraviolet-visible spectrophotometer (490 nm) [18,19].

### 2.5 Statistical analysis

All the data of cytotoxicity activity were presented as the average of triplicate analyses. The outcomes were presented as means ± standard deviation (SD).

## 3 Results and discussion

### 3.1 Chemical composition of curcumin

**Figure 1:**
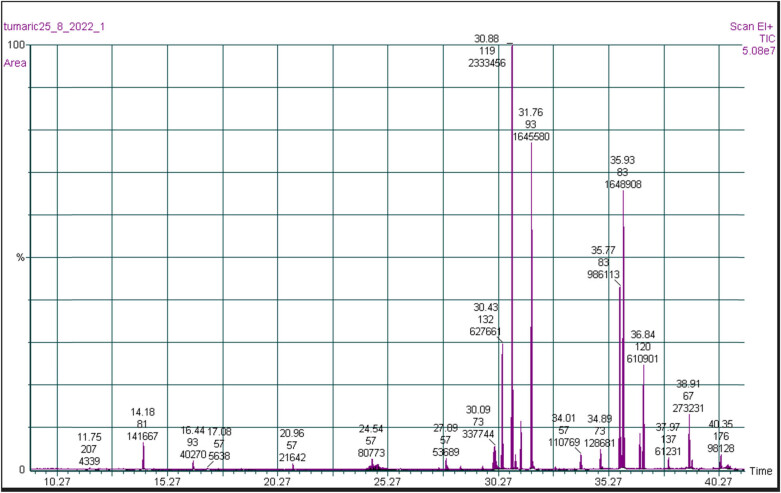
GC–MS of *Palestina Turmeric rhizome* extraction.

A GC–MS combination was used to determine the chemical composition of the curcumin plant’s extracts on both a qualitative and quantitative level (Figure 1). The identities and amounts of the chemical components isolated from curcumin are presented in Table 1.

**Table 1:** Chemical composition of *C. longa* rhizome extract

Chemical constituent	R.T (min)	K.R.I	L.R.I	Area	% content ± SD
Eucalyptol	14.21	1030.353	1,030	141,667	1.67 ± 0.65
Terpinolene	16.43	1082.588	1,082	40,270	0.47 ± 0.01
α-Curcumene	30.44	1473.054	1,473	627,661	7.40 ± 0.12
α-Zingiberene	30.88	1492.308	1,492	2,333,456	27.51 ± 1.77
β-Bisabolene	31.284	1500.143	1,500	248,510	2.93 ± 0.12
β-Sesquiphellandrene	31.76	1517.204	1,517	1,645,580	19.40 ± 0.14
Ar-tumeron	35.77	1660.932	1,660	986,113	11.63 ± 0.24
Tumeron	35.95	1667.384	1,668	1,648,908	19.44 ± 0.59
Germacron	36.67	1693.19	1,693	199,584	2.35 ± 0.06
Curlone	36.85	1699.642	1,699	610,901	7.20 ± 0.18
Sum				8,482,650	100.00

The total plant is comprised of ten compounds, and most of the characterized metabolites are terpenoids and oxygenated terpenoids. As can be seen from Table 1, α-zingiberene is the major component (27.51%), followed by tumeron (19.44%), β-sesquiphellandrene (19.40%), and aromatic tumeron (11.63%). Mau isolated EOs from the rhizomes. They isolated a total of 36 compounds but were only able to structurally characterize epicurzerenone and curzerene [20]. In a similar study by Rahman et al., screening of the chemical compositions of turmeric rhizomes was accomplished by introducing both the raw and methanol extracts to mass spectrometry in positive and negative modes. The mass spectra results of the analyzed raw turmeric rhizomes could identify 13 bioactive compounds including curcumin [21].

### 3.2 HPLC results

**Figure 2:**
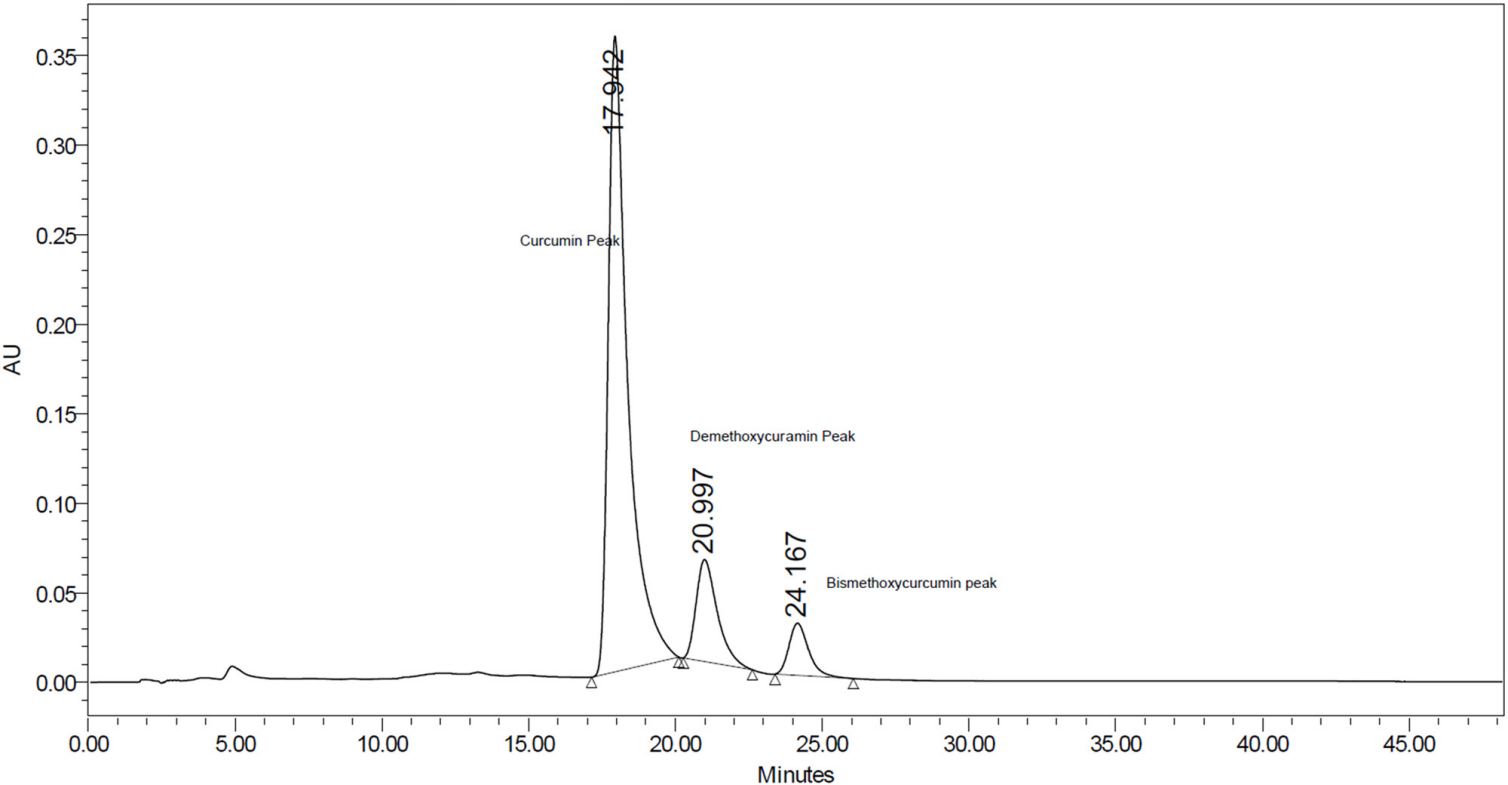
The chromatogram of the tested extract of *C. longa* plant rhizomes.

Overall, the HPLC analysis allowed for the identification and quantification of curcumin and its derivatives in the plant extract, providing valuable information about their relative proportions. The results from the HPLC chromatographic conditions demonstrated that well-separated peaks of curcumin and its derivative were obtained, as shown in Figure 2. The area under the curve of the HPLC-eluted peaks provided information about the relative amounts of curcumin and its derivatives in the plant extract.

According to the analysis, the curcumin present in the plant extract accounted for 1.87% of the total weight of the extracted oil. This implies that the remaining percentage represents other curcumin derivatives. It is likely that the HPLC analysis revealed additional peaks corresponding to these derivatives, indicating their presence in the extract.

The empirical formula and the percentages of the derivative in the examined extract by HPLC are shown in Table 2.

**Table 2:** Analysis of DCM extraction of *Palestina Curcuma longa* L. root by HPLC

Curcumin derivatives name	Empirical formula	Percentage
Curcumin	C_21_H_20_O_6_	79
Demethoxycurcumin	C_20_H_18_O_5_	13.81
Bisdemethoxycurcumin	C_19_H_16_O_4_	6.45

**Figure 3:**
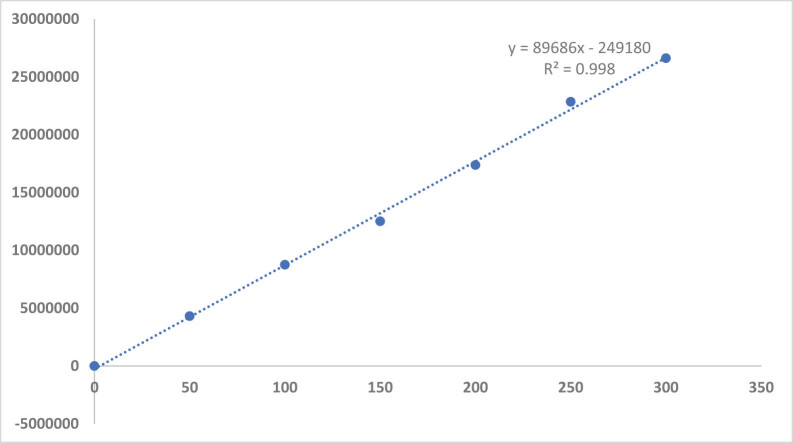
The calibration curve of a serial standard of curcumin.

The chromatograms of the used HPLC method showed linearity. The areas of the standards were plotted against its concentration, and the generated calibration curve and the regression line was: *y* = 8,9686*x* – 249,180 with square correlation coefficient (*R* ^2^ = 0.998) demonstrating high degree of linearity (Figure 3).

A similar study conducted by Opustilova et al. also performed an HPLC analysis of curcumin and reported three peaks. The main peak corresponds to curcumin itself, while the other minor peaks were observed near the major peak of demethoxycurcumin and bisdemethoxycurcumin. This suggests that curcumin derivatives, such as demethoxycurcumin and bisdemethoxycurcumin, were detected in their study as well [22,23,24].

**Figure 4:**
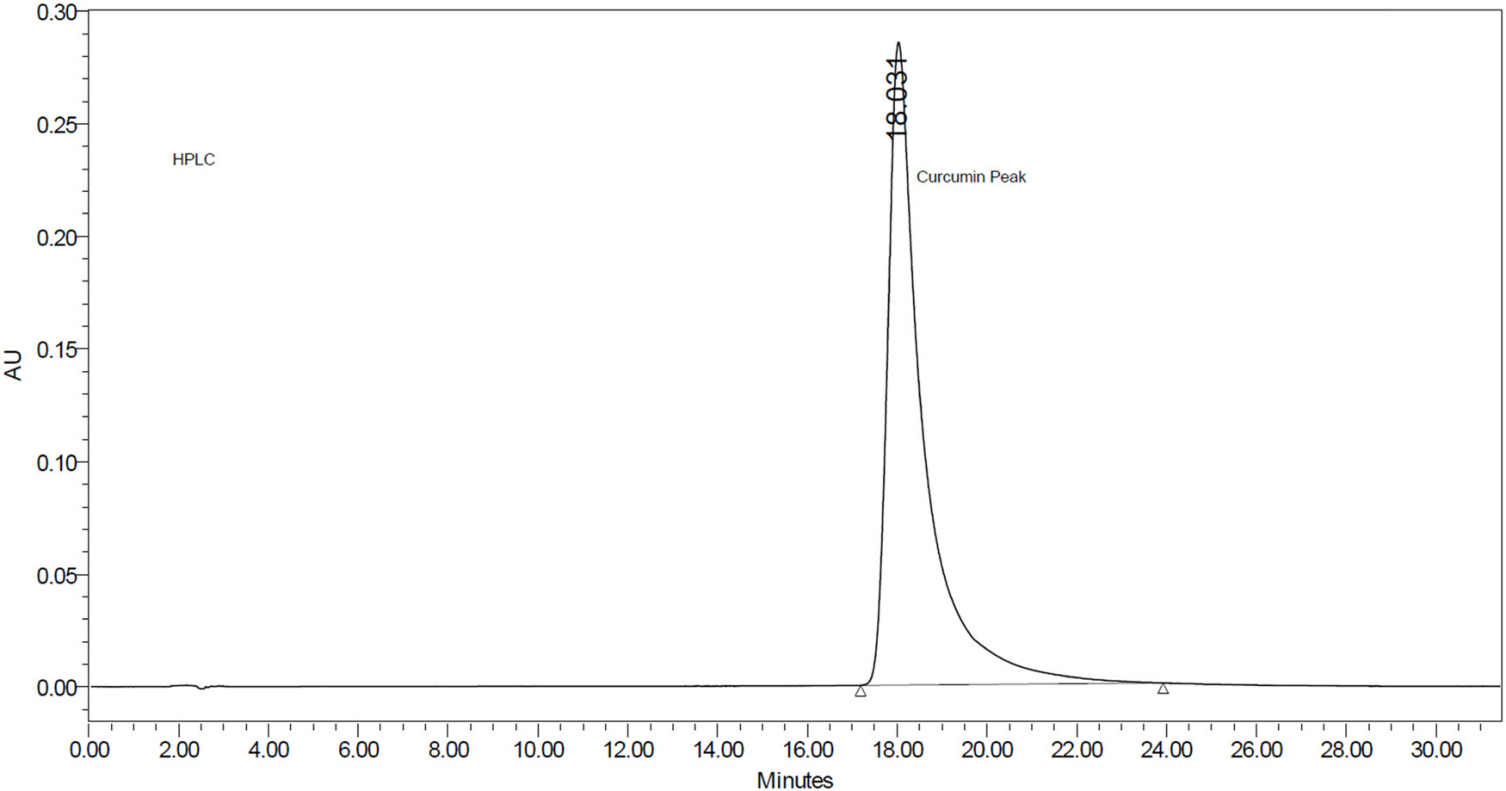
HPLC chromatogram of the curcumin reference standard.

The applied HPLC method showed a resolution of the eluted peak of curcumin and its derivatives. Moreover, the eluted peaks of the chromatograms were of high theoretical (>2,000) plates and acceptable symmetry. An example of the eluted peak of the curcumin standard is shown in Figure 4.

The calculated limit of detection and quantification of the applied method was 2.3 and 8.2 µg/mL, respectively.

The results of this study were comparable with other similar work done in the literature. Garg et al. studied the *C. longa* that was grown in the climatic conditions of the North Indian plains at Lucknow; they studied the extracted oil and tested it for its major terpene components including curcumin. The result of the study showed the sum of the seven major terpenes in the range of 58–79% [25]. Setzer et al., in their study of five different varieties of *C. longa*, which were cultivated in north Alabama, showed that curcumin constitutes around 6.8–32.5% of the total extracted oil [26].

### 3.3 Cytotoxicity

The *C. longa* L. root extract was tested against various cancer cell lines, including hepatocellular carcinoma (Hep 3B), skin tumors (B16-F1), cervical adenocarcinoma (HeLa), colorectal adenocarcinoma (COLO 205, Caco-2), and human hepatic stellate (LX-2) cells. The results indicate variation in inhibition activity according to cancer cell line type. The results illustrate moderate to potent activity against HeLa and LX-2 cancer cells. The detailed IC_50_ results are shown in Table 3.

**Table 3:** IC_50_ result of the Palestina *Curcuma longa* L. root on different cancer cell lines

Tests	IC_50_ (µg/mL)			
	HeLa	B16F1	Hep3B	LX-2
Curcumin	30.25 ± 0.2	52.48 ± 3.55	46.84 ± 2.12	29.77 ± 1
Doxorubicin	5.24 ± 1.022	≫0.05	0.434 ± 0.271	≫0.05

**Figure 5:**
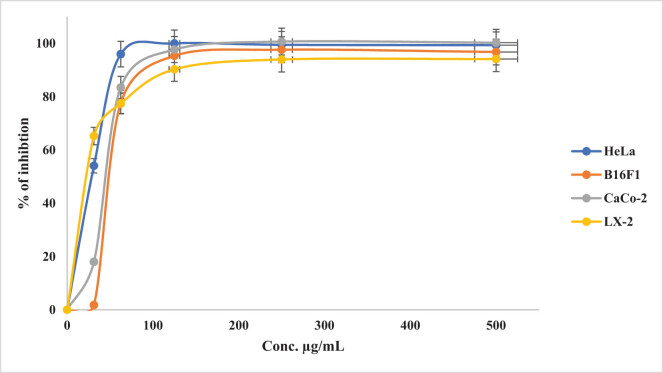
Percentage inhibition of *C. longa* L. root extract (0–500 µg/mL) on four cancer cell lines.

**Figure 6:**
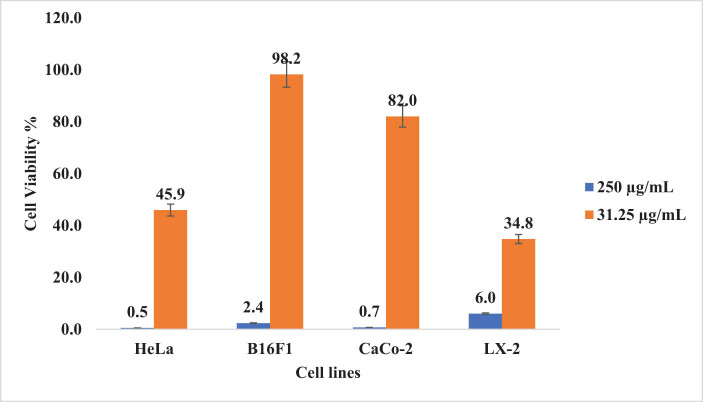
Cell viability results of four cancer cell lines treated with two concentrations (31.25 and 250 µg/mL) of *C. longa* L. root extract.

The cytotoxicity results clearly demonstrated the potent inhibition activity of curcumin against HeLa cancer cells. Moreover, a higher concentration of the plant (0.5 mg/mL) caused more than 90% inhibitions of all the tested cancer cells (Figure 5). The plant extract showed a significant increase in inhibition on all cancer cell lines when the concentration was increased from 31.25 to 250 µg/mL. The results illustrated in Figure 6 demonstrate that no more than 10% of viable cancer cells in all the examined cell lines were observed at a concentration of 250 µg/mL.

Curcumin has shown promising potential as an anticancer remedy due to its various activities, which can act both in a chemopreventive manner and directly as a therapeutic agent. While some of these effects have been observed in animal models, curcumin has also demonstrated activity in numerous *in vitro* models [27,28]. Many studies have demonstrated that curcumin induces cell death in a wide range of animal and human cell lines, including those associated with leukemia, melanoma, and various carcinomas. Numerous *in vitro* studies have demonstrated the cytotoxic effects of curcumin on cancer cells derived from different organs, including the breast, lung, colon, kidney, ovaries, and liver. Our data are consistent with previous studies that reported curcumin exerts its anticancer effects via proliferation inhibition and apoptosis induction in various cancer cell lines [29,30,31,32].

Plant chemical composition is affected by climate, temperature, rainfall, soil type, and sunlight. These factors can alter the synthesis and concentration of bioactive secondary metabolites such as phenolic compounds, alkaloids, and terpenoids. Turmeric rhizomes contain polyphenolic curcumin. A region’s climate and environment affect turmeric’s curcumin content. Turmeric grows best in warm regions, which increases curcumin concentration in the rhizomes. Turmeric needs enough rain. Stress from drought or abundant rains might lower curcumin levels. Soil pH and nutrient levels affect curcumin content. Turmeric plants need sunshine for photosynthesis and curcumin production. Cytotoxicity can vary with ambient curcumin levels. Turmeric with higher curcumin concentrations may have stronger cytotoxic effects in cancer research and treatment [33,34,35]. The favorable climate conditions in Palestine, with adequate sunlight and rainfall, can contribute to the growth of turmeric plants with a relatively high polyphenolic curcumin content. This explains the potent cytotoxicity results in this research work.

## 4 Conclusion

The current research is the first of its kind to explore the phytochemical and anticancer activities of this plant species found in Palestine. The current results revealed the presence of many phytochemicals in the extracts of *C. longa*. Moreover, the HPLC analysis showed separated peaks of curcumin and its derivatives. The plant extract showed potent anticancer activity against Hela and LX-2 cancer cells. The cytotoxicity test also showed that the extracts have cell inhibition activity of more than 90% for all the tested cancer cell lines at a higher concentration of 250. These findings indicate that *C. longa* collected from Palestine is a promising natural source of potent anticancer activity. In fact, it can be used in future pharmaceutical formulations and as a treatment strategy for cancer diseases.
